# Digitising biopiracy? The global governance of plant genetic resources in the age of digital sequencing information

**DOI:** 10.1080/01436597.2022.2079489

**Published:** 2022-06-03

**Authors:** Ryan Nehring

**Affiliations:** Department of History and Philosophy of Science, University of Cambridge, Cambridge, UK

**Keywords:** Agricultural research, open source, biotechnology, plant treaty, CBD, agriculture and food security

## Abstract

Historical concerns over the exploitation of the Global South’s genetic biodiversity framed the importance of creating global governance mechanisms to ensure fair access to and benefit-sharing of genetic resources worldwide. The Convention on Biological Diversity (CBD) and International Treaty on Plant Genetic Resources for Food and Agriculture (Plant Treaty) came into existence over the past three decades to redress the centuries of genetic exploitation of the Global South. Both of the treaties explicitly regulate and facilitate the exchange of physical genetic material. The recent emergence of relevant digital technologies, such as digital sequencing information (DSI), could make both treaties irrelevant. This article analyses the current state of the CBD and Plant Treaty as it relates to global agricultural research in light of DSI. I argue that DSI presents less of a threat to exacerbating historical gene flows than it does to the further displacement of public sector research by the private sector. The article then suggests looking at the lessons from open-source approaches to counter the privatisation of DSI and related gene flows. I draw on 11 key informant interviews with country negotiators involved with the CBD and Plant Treaty as well as a review of official reports from both frameworks.

## Introduction

In 1876, Henry Wickham arrived in the Brazilian Amazon under contract with the Royal Botanic Gardens at Kew in London. He collected around 70,000 rubber seeds which were highly perishable due to their moisture sensitivity as so-called ‘recalcitrant seeds’.^1^ Wickham’s ability to successfully transport the rubber seeds across the Atlantic was made possible due to his spending years working alongside locals to learn about the natural conditions required for rubber seeds and rubber plants to survive (see Jackson 2008). When his seeds were finally germinated in the UK and then successfully transported to the tropics in Southeast Asia, the Brazilian monopoly on rubber collapsed. Brazil’s share of the global rubber supply plummeted from 95% at the end of the nineteenth century to less than 5% by the mid 1920s. Brazilian officials vilified Wickham, whose collection of rubber seeds was later considered to be the ‘original act of biopiracy’ (Gollin 2008: 1055).

With the collapse of formal European colonialism throughout the nineteenth and twentieth centuries, newly independent countries moved to establish sovereignty over their genetic resources. Genetic diversity was a hopeful source of national wealth as much as it was a national security issue, as in the case of Brazilian rubber. Centuries of social domination and ecological extraction under colonialism resulted in a legacy of deeply unequal exchanges of people and plants. Historians have labelled this historical trend as a ‘south–north gene drain’ or ‘Columbian Exchange’ (Crosby 2003), where biologically diverse regions of the ‘New World’ were violently exploited for their richness in genetic diversity. These forms of unequal exchange in genetic material are also intrinsically linked to unequal relationships of development (Mooney 1983). The term ‘biopiracy’ is attributed to the scholar-activist Pat Mooney, who argued that trade agreements and existing multilateral organisations exacerbated existing inequalities in (genetic) resource access and control between the Global North and Global South. Biopiracy, then, is both a descriptor for gene flows and a claim of global (in)justice (Mooney 2000; see also Robinson 2010; Merson 2000).

One of the common narratives of the South–North gene flow is that the majority of the world’s biodiversity is located in the Global South but it is the Global North that maintains the technological capacity and political power to exploit it economically (eg Halewood, Noriega, and Louafi 2013; Kloppenburg 2004). Early modern techniques of transporting plant genetic material were particularly inefficient and unequal. Transoceanic passages under sail presented treacherous conditions for all living organisms. European governments invested in new technologies that could increase the survivability of plant genetic resources during the extraction process. Parsons and Murphy (2012) cited Royal Society fellow John Ellis who, in 1770, claimed that ‘in most cases fewer than one plant in fifty might survive the [transatlantic] trip’ (504). Put another way, power imbalances that shaped the flow of plant genetic material were inherently linked to knowledge on *and* technological capacity over the biological barriers of transcontinental travel.

A more recent example commonly used to support the narrative of genetic exploitation in the Global South is the ‘shuttle breeding’ programmes during the Green Revolution in the middle of the twentieth century. These programmes took advantage of participating governments and farmers in the Global South to rapidly breed varieties of grain crops in new environments (Pingali 2012). While the purported benefits of the Green Revolution for poor farmers have largely fallen short (see Patel 2013), scientific institutions and industries in the Global North undoubtedly benefitted from the territorial and ecological expansion of agronomic experimentation in the tropics (Raby 2017; Harwood 2018).

Over time, the process of improving plants has increasingly been decoupled from their place of origin. Recent trends in gene flows for food and agriculture have reversed: the Global South is now a net importer of improved plant varieties (Fowler, Smale, and Gaiji 2001). Nevertheless, the historical narrative of biopiracy and genetic exploitation continues to shape the policy debate on how to best govern the access to and benefit-sharing of plant genetic resources. The conservation of genetic biodiversity is currently being framed by policymakers as precisely the reliance on a robust exchange of genetic resources, only under more favourable terms for the Global South.

The first major international effort to govern the exchange of genetic material was the Convention of Biodiversity (CBD) of 1992. The CBD was based on the idea that interdependence of genetic resources was the most environmentally sustainable and politically desirable way to conserve global genetic biodiversity for the future. Article 2 of the CBD defines genetic resources as ‘genetic material of actual or potential value’. Benefit-sharing under the CBD was later established under the Nagoya Protocol ‘to ensure that the *physical* access to genetic resources is facilitated and that the benefits obtained from their use are shared equitably with the providers’ (CBD 2010, 4, emphasis added). In other words, the active use and exchange of genetic resources ensure the conservation of genetic diversity across space, but equitable terms of benefit-sharing are established by recognising its place of origin. As Bond and Scott (2020) put it, the Nagoya Protocol and the CBD ‘territorialise’ the access to and benefit-sharing of genetic resources for food and agriculture. The territorialisation of genetic resources fixes the place of origin to govern the access and benefit-sharing for any interested party.

Benefit-sharing has been hotly contested since the inception of the CBD almost three decades ago. The Nagoya Protocol was envisioned to be part of the solution to redress historical trends of material genetic transfers from the Global South to the Global North. However, despite ongoing negotiations, the transfer of genetic resources has been rapidly digitising: physical access to genetic material is being superseded by the increasing availability of digital sequencing information (DSI). A digital future for the exchange of genetic resources presents new challenges for the ways in which historically exploited ‘gene-rich’ countries can implement mechanisms based on the fair and equitable sharing of benefits. In particular, the determination of ‘origin status’ for any sample is increasingly difficult as databases lack traceability mechanisms and the cost of gene sequencing is rapidly decreasing. Importantly, the CBD explicitly states that it applies only to ‘physical access of genetic resources’ and not ‘genetic information’ or a similar broad term. This wording has caused significant confusion among policymakers and the Contracting Parties of the CBD regarding whether and under what conditions DSI is included within the CBD governance framework (see Cowell et al., 2022). Therefore, this article seeks to answer: how are emerging technologies shaping the terms of global governance over genetic resources for food and agriculture and what are the implications for both public and private agricultural research?

Recent works by scientists on the global governance of genetic exchange view DSI as an instrument to facilitate the creation of a ‘global commons’ of genetic resources (Bruynseels 2020). They argue that the ‘unlimited and open access’ of DSI will help foster collaboration and innovation that will better serve the goals of biodiversity conservation (Prathapan et al. 2018). Their support of open DSI stands in stark contrast to the governance mechanisms, such as the CBD, that were implemented to establish a framework of access and benefit-sharing to serve countries in the Global South. The emergence of DSI, as a broad set of tools to more quickly and openly share genetic resources for food and agriculture, has pitted those in favour of using the technology as a means to create a common pool of resources against those who continue to argue for redressing centuries of genetic exploitation.

I argue that DSI presents less of a threat to exacerbating historical gene flows than it does to the further displacement of public-sector agricultural research by the private sector. The private sector has expanded its reach over global genetic resources for food and agriculture while public spending has decreased (Fuglie and Toole 2014). Furthermore, the emergence of a global intellectual property framework since the 1990s has bolstered the worldwide demand for genetic resources as the central raw material for biotechnology companies. Liberalised trade benefits private-sector research, whereas the public sector still relies primarily on the governance mechanisms under the CBD and Plant Treaty. The result is a contradictory and contested system that disproportionately constrains the public sector (Görg and Brand 2006). I suggest that lessons can be taken from open-source approaches in agricultural research that aim to improve access to and the conservation of genetic resources for all.

This article starts with an historical overview of the governance frameworks to oversee the sharing of genetic biodiversity for food and agriculture at the end of the twentieth century. It was in the 1990s that governments negotiated the terms of the CBD and International Treaty on Plant Genetic Resources for Food and Agriculture (hereafter Plant Treaty) as the primary governance mechanisms for the sustainable access to, and use and benefit-sharing of, global genetic resources for agricultural research. The next section introduces the recent explosion and controversy of DSI as an emerging set of technological tools, as well as the challenges DSI poses for the CBD and Plant Treaty. Lastly, this article will offer the example of open-source seed networks as a ‘protected commons’ approach that provides one potential avenue to safeguard DSI tools for global public agricultural research.

The research presented in this article draws on 11 semi-structured key informant interviews with plant breeders and Contracting Party negotiators involved in the CBD and Plant Treaty. All of the interviews were conducted in March or April 2021 via virtual conferencing software. Eight of these interviews were with professionals based in or representing countries in the Global South. Three interviews were with scientists working within the global governance framework who have been active in arguing for reforms to both the CBD and Plant Treaty as it relates to DSI. The interviews were conducted to provide information on the motivations and strategies of Contracting Party negotiators, while interviews with the scientists revealed how they work within the CBD/Plant Treaty rules and how they utilise DSI on an everyday basis for their research. This research also draws significantly on a review of expert reports from the CBD and Plant Treaty governing bodies.

## The digital challenge of genetic resource governance for food and agriculture

The treatment of genetic resources as a static concept of nature to be collected, catalogued and then conserved emerged at the end of the nineteenth and into the early twentieth century. Bonneuil (2019) calls this logic ‘genetic modernism’, which understands biological diversity to be a set of static units at the gene level that ‘co-produce a new global kind of knowledge and a new geopolitical object’ (12). Seed banks then became the primary institutions to deploy this understanding of genetic diversity in the twentieth century. According to Curry (2017), seed banks were, and continue to be, technologies for the ‘maintenance of genetic diversity’ that are part and parcel of the uniformity of the field under industrialised agriculture. Under genetic modernism, the field is considered a site of genetic uniformity, while seed banks are organised sites of genetic diversity. International networks between governments and philanthropic organisations, such as the Rockefeller Foundation, helped to establish the most important seed banks and agricultural research centres under the Consultative Group for International Agricultural Research (CGIAR) (Byerlee and Lynam 2020). Agricultural development programmes directed at the Global South in the middle of the twentieth century drew upon these networks to understand and collect crop diversity while at the same time promoting productive uniformity of a select high-yielding varieties of major commodity crops.

In 1974, the International Board for Plant Genetic Resources was established to manage and regulate genebanks around the world. National governments argued over the best way to manage global genetic resources with claims that the US was not making materials available under the International Board (Mooney 1983; Thormann, Engels, and Halewood 2019). At the heart of the debate was how and the extent to which benefits from genetic resource use could be shared with countries of origin. The so-called ‘seed wars’ pitted the Global South against the Global North as governance mechanisms were seen as the way to redress centuries of colonisation and the concomitant gene flows. The seed wars then set the stage for the battle over the governance of genetic resource access and benefit-sharing in the ensuing decades.

It was only in 1992 at the United Nations Conference on Environment and Development in Rio de Janeiro, Brazil that the CBD was established by 196 countries to consider genetic resources as under national sovereignty. Just over a decade later, in 2014, the Nagoya Protocol of the CBD established international access to and the sharing of benefit from genetic resources in accordance with prior informed consent of the country and under mutually agreed terms. The Nagoya Protocol was specifically the result of concerns from countries in the Global South that felt the CBD was incomplete by not ensuring compliance with Access and Benefits Sharing (ABS) (see Greiber et al. 2012; Richerzhagen 2014). For example, under the CBD, benefits accrued from the commercial use of genetic resources ‘may include monetary and non-monetary benefits’ in accordance with domestic laws that govern access and benefits sharing (CBD 2011, 6). Negotiations over the Nagoya Protocol took some 18 years of intense debate between Contracting Parties in order to agree upon how to structure ABS. There was a divide between countries that consider themselves to largely be ‘providers’ of genetic resources, or those mainly in the Global South, and ‘users’, which are mainly in the Global North. The Nagoya Protocol has been ratified by 128 parties and addresses issues of compliance with and the enforcement of ABS within bilateral agreements.

Some have argued that the Nagoya Protocol prompted national governments to adopt more restrictive and time-consuming legal regimes that limit access to their genetic resources (Prathapan et al. 2018). In other words, without a multilateral system, national protectionism would limit access to and the use of genetic resources and, most importantly, the interdependence of conserving genetic biodiversity (Scholz et al. 2022). Thus, efforts were undertaken to treat plant genetic resources as a shared global heritage rather than under the principle of national sovereignty.

Multilateralism is at the heart of the Plant Treaty, which states that plant genetic resources are a ‘common concern of all countries’, but that efforts to support ‘the capacity of developing countries and countries with economies in transition’ need to be ‘urgently enforced’ (ITPGRFA 2004, V). The Plant Treaty was signed by 147 countries and has been in effect since 2004. It operates in parallel to the CBD with ostensibly similar aims to promote the sustainable use and sharing of genetic resources; however, it is specific to plant genetic resources for food and agriculture. Article 2 of the Plant Treaty defines ‘plant genetic resources’ as ‘any material of plant origin, including reproductive and vegetative propagating material, containing functional units of heredity’. The legal scope of the Plant Treaty only applies to a list of 64 crops. This list, called the ‘Annex I crops’,^2^ consists of 64 food and forage crops that were hotly debated by countries since the negotiations started in the late 1990s. The core of the Plant Treaty is what is called the Multilateral System (MLS) of access and benefit-sharing of plant genetic resources. The final list of Annex 1 crops was crucial to the MLS because it would move beyond the ‘country of origin’ status that is at the heart of the CBD. Under the MLS, all of the 64 Annex I crops are considered a global common pool of material genetic resources, albeit a limited one.^3^ Some of the strongest early proponents of an MLS were international seed banks and research centres that wanted to promote a global framework to govern the sharing, use and benefits of crops based on their experience of international collaboration (see Visser 2013). Indeed, the Plant Treaty is used almost exclusively by public research institutions. Further, over half of all plant genetic material used by plant breeders from national research programmes around the world came from public organisations (25% from the Global North and 28% from the Global South) (FAO 2010, 96).

Taken together, the CBD (including the Nagoya Protocol) and Plant Treaty provide a substantial advance in the governance of genetic resources worldwide. However, they only apply to material, and not necessarily digital, genetic resources. And the MLS only applies to a select 64 crops. Perhaps most importantly, the Plant Treaty does not have legally binding contracts to enforce payments from private-sector users (Tvedt 2021). All of these shortcomings in reach and scope have led to significant criticisms on the relevance of the Plant Treaty in practice (Noriega, Wambugu, and Mejías 2013; Mwila 2013; Rabitz 2017). It is perhaps not surprising then that the recent advances in gene sequencing technology are threatening to make both the Nagoya Protocol and the Plant Treaty irrelevant.

The overarching historical narrative behind the development of the CBD and Plant Treaty was based on redressing genetic flows from the Global South to the Global North. Despite the limitations of both frameworks, they do provide a means through which access and benefit-sharing of genetic resources can be negotiated. Nevertheless, there is a fear amongst the Contracting Parties of the two agreements that the dematerialisation of genetic resources, facilitated by DSI, will completely sideline any further negotiations or the relevance under a digitised world of genetic resource sharing. This presents an obstacle for negotiating parties to now define DSI and how it can be integrated within the existing governance frameworks. Recent scholarship on the question of DSI as it relates to both frameworks has determined that the original agreements themselves have to be reimagined if they are to maintain relevance in this new digital age of genetic resources (cf Bond and Scott 2020; Aubrey et al. 2021).

## Defining and governing DSI

DSI is a ‘placeholder’ term that is used by the CBD, Plant Treaty other international agreements because no agreed-upon definition has been established (Houssen, Sara, and Jaspars 2020). Thus far, the CBD Governing Body has been leading the discussions on how to define DSI and what role it can and should play in shaping global gene flows. There are ongoing discussions around the extent to which different genetic markers should be included under the definition of DSI or perhaps even using an alternative term altogether. Recommendations thus far have called for the core nucleotide sequence data (DNA and RNA) to fall within the ‘narrow’ category of DSI, with subsequent categories adding proteins, metabolites and traditional knowledge, respectively. The more limited definition of DSI to include nucleotide sequence data allows for breeders to know the location, role and structure of DNA within a given genetic resource. Therefore, DSI is less of a specific tool as it is a catch-all for a range of both data and tools that have far-reaching applications in areas such as medicine, agriculture and energy. To narrow the scope of DSI, this discussion focuses primarily on the implications and practice of DSI for agricultural research.

The theory of genetic sequencing information has been around ever since Francis Crick worked on the ‘Central Dogma of Molecular Biology’ – the sequence of DNA to RNA to proteins in molecular biology – in the late 1950s (Crick 1970). However, sequencing information has only become more widely applied in biology over the past couple of decades. In practice, nucleotide sequence data commonly rely on the use of subsidiary information such as protein sequences and metabolites (Houssen, Sara, and Jaspars 2020). The addition of traditional knowledge or accompanying phenotypes can also be useful for any plant breeders who utilise DSI. This makes any categorisation difficult for a number of reasons. For one, benefit-sharing through a more narrow categorisation will limit the utility and, ultimately, the use of said DSI. However, including all relevant information (ie traditional knowledge or place of origin) under the definition of DSI would create a more expansive categorisation that is inconsistent with existing data. Not all DSI includes accompanying categories (ie traditional knowledge), and there are significant barriers to traceability.

One of the most common uses of DSI in food and agriculture is to assess and quantify the level of genetic variation between accessions at and between *ex situ* and *in situ* genebanks. The International Potato Center in Peru recently used DSI to generate a genotype profile for all of the accessions at their genebank. Similar uses of assessing genebank genotype diversity have been deployed for ‘creole wheat’ at the International Maize and Wheat Improvement Center (CIMMYT) in Mexico and in Africa and with landrace rice varieties at the International Rice Research Institute (IRRI) in the Philippines (see Halewood et al. 2017). In this sense, DSI can be effectively used more as a tool for genebank managers to ‘rationalise’ accessions such that they adequately represent desirable biodiversity and minimise duplication. Prior to the use of DSI in seed banks, curators struggled to manage vast amounts of data – both physical documents and databases – that referenced physical accessions and associated information (ie characteristics, traits, origins, etc.) in the banks. In addition to applications in genebanks, DSI is also being used in the new wave of biotechnology. Novel gene editing methods such as CRISPR (clustered regularly interspaced short palindromic repeats) are rapidly being developed to allow plant breeders to use genetic markers as a guide for trait-level changes in individual genomes. DSI is necessary for CRISPR so that plant breeders can locate which genes are desirable for editing.

Beyond the current applications of DSI, there are also perceived uses that are feared by the governing body of the CBD and Plant Treaty. Synthetic biology is an area of research that attempts to create or redesign nature without relying on natural inputs. In the food and agricultural sector, researchers have successfully synthesised natural enzymes such as sweeteners and even plant functions such as nitrogen fixation and photosynthesis (see Wurtzel et al. 2019; Roell and Zurbriggen 2020). Synthetic biology threatens to remove the origin status of many plants, which undermines the original principles of sovereignty established by the CBD (see CBD 2015). And the perceived further uses of synthetic biology are also viewed by the Plant Treaty Governing Body as a viable method to bypass the need to access to physical genetic resources from the MLS (Aubrey 2019).

The issue of DSI is being hotly debated between the Contracting Parties of the CBD and Plant Treaty. In 2018, the CBD appointed an Ad Hoc Technical Expert Group on Digital Sequencing Information on Genetic Resources to gather data on the issue of DSI as it relates to global governance issues on genetic resources. However, the debate remains at a standstill as of early 2022. One country negotiator stated that DSI is exacerbating negotiations because ‘the CBD has a very big weakness in terms of multinational governance in that the users and providers don’t have the same terms’ (personal interview, 24 March, 2021). In their terms, the ‘users’ are largely countries in the Global North whereas the ‘providers’ are Global South countries. Although the CBD and Plant Treaty were established precisely to redress historical transfers of genetic resources from the Global South to the Global North, DSI is threatening to circumvent the access and benefit-sharing tools that have been established since the Nagoya Protocol.

Downstream effects of DSI are also involving other global governance frameworks. The World Intellectual Property Organization (WIPO) is working with the CBD and Plant Treaty to assess the role of DSI in issuing patents that utilise genetic resources and are subject to access and benefit-sharing. For WIPO, the issue of DSI has been increasingly involved in the debate over whether and how patentability can be claimed when traditional knowledge (TK) is involved (see Farhat 2008). As Smyth et al. (2020) put it,

if an indigenous group [*sic*] has reared a particular plant to express desired traits over generations, if said plant’s genome is sequenced, embedded within this DSI is the TK of the indigenous group that reared the plant. The question, therefore, for those developing DSI governance is whether and how the ‘information’ in DSI is distinguishable from the ‘knowledge’ in TK. At present, there does not seem to be a feasible way to legally or politically dissociate TK from the DSI of a sequenced plant. (276)

WIPO has been involved in determining how and the extent to which benefits can be shared under TK terms once patents are potentially issued downstream (see WIPO 2018). As will be explained in the next two sections, downstream patenting poses specific risks to ensuring an open-source data infrastructure of DSI.

Technological advances in DSI have outgrown the CDB and Plant Treaty, and the topic of DSI is now overrunning the ongoing negotiations involved in ratifying them. Not only is the country of origin status complicated by the broad trend in the digitisation of genetic resources but, through synthetic biology and other uses of DSI, it is becoming completely irrelevant. Nevertheless, DSI infrastructure is largely available under open-access conditions, despite being managed and funded primarily by only a few countries. The CBD and Plant Treaty were envisioned to shift the tide in the historical transfer of genetic material from ‘gene rich’ countries to ‘gene poor’ ones (Fowler et al. 2001). But now the digitisation of genetic resources for food and agriculture is threatening to erase any gains that have been made thus far. Attempts to include DSI and synthetic biology within the CBD and Plant Treaty are now dealing with questions of ownership over the original genetic material and the existing infrastructure of DSI databases. The actual and perceived uses of DSI are far-ranging and beyond the scope of analysis for this paper (see Aubrey 2019; Houssen, Sara, and Jaspars 2020; Halewood et al. 2018; Heinemann, Coray, and Thaler 2018). However, it is key to situate the production, storage and use of DSI databases as a lens through which we can analyse questions of ownership/open access over DSI data.

## DSI infrastructure and ownership

The availability of DSI has exploded in recent years, especially in the food and agriculture sector. Part of the reason for this increase in data is the rapid decline in costs associated with genome sequencing. For example, in under two decades, the cost to sequence a human genome fell from $100,000,000 in 2001 to around $1000 in 2020.^4^ The largest depository and source for DSI is the International Nucleotide Sequence Data Collaboration (INSDC). The INSDC is the culmination of a collaborative multi-country effort to consolidate three existing DSI databases: the GenBank in the US, the European Nucleotide Archive (ENA) in the UK and the DNA Data Bank of Japan. What forms the INSDC is the ‘mirroring’ of all shared DSI among each of these three databases every 24 hours to maintain a current copy of all published data available between the three existing databases. Of all the other DSI databases in the world, 95% are directly linked to or download their data from the INSDC (Smith 2020). The collaboration is based on the principle that DSI is ‘permanently accessible’ with ‘free and unrestricted access’ worldwide. However, the INSDC and its database partners maintain a hands-off approach concerning the underlying restrictions or copyrights associated with any of the sequence data. Data submissions are encouraged to be made anonymously to maintain INDSC’s distance between data ownership claims and the users (see Lawson and Rourke 2016). In other words, ‘INSDC databases are data hosts and not data owners’ (Cochrane et al. 2011).

The total cost to operate the INDSC is estimated at $50–60 million a year (Smith 2020). Data is not available on the distribution of those costs between the managing countries. But all of the countries are in the Global North (US, UK/Europe and Japan). Prior to the INSDC, plant breeders specifically faced constraints on access to data due to the lack of an overarching model or meta-database (Leonelli et al. 2013). Rohden et al. (2020) estimate that approximately 76% of the almost one million sequence entries held by the INDSC are relevant to the CBD framework. There are also agriculture-specific DSI databases, which are highly concentrated within the Global North. For example, the US Department of Agriculture houses GrainGenes, MaizeGDB, SoyBase and the Legume Information System. Tthese databases are federally funded by the US government and operate under the same open-access principles (*libre*), with over 100,000 users annually (see Gaffney et al. 2020).

The ongoing discussions in the Governing Bodies of the Plant Treaty and CBD over the definition of DSI also has implications for traceability. A narrower definition would facilitate the creation of ‘place of origin’ data that could then be used for claims of sovereignty over DSI (ie CBD) and/or the ability to add a multilateral system for DSI (ie Plant Treaty). One or both of those options could also fit within different definitions of DSI (see WiLDSI 2020). For example, a narrow definition of nucleotide sequencing data could potentially apply to the CBD modality of national sovereignty over genetic resources, whereas protein sequencing, metabolites and traditional knowledge could fall under the Plant Treaty. In such a case, distributing different components of DSI into two different frameworks would then require new mechanisms for data management and traceability. Yet only 16% of all data entries in the INDSC have a country of origin listed, with one-third of those entries originating in either China (18%) or the US (17%). A traceable connection to a publicly available material genetic resource in a collection (eg botanical garden, seed bank) is even more scarce, at 6% of total entries (Rohden et al. 2020). The reason for this lack of traceability is the fact that anyone can submit sequence data and there is no requirement for users to list the origin of their submission.

There is much better data on the user location of DSI from the INDSC. China and the US represent around 40% of the users of the database, which are estimated at as much as 500 million people worldwide (Rohden et al. 2020). Clearly, problems of traceability and transparency need to be overcome if any benefit-sharing framework, whether bilateral (ie CBD) or multilateral (ie Plant Treaty), intends to govern DSI. Germany’s WiLDSI (2020) proposed a series of options intended to find a middle ground between open access and tracing origin and users of DSI for benefit-sharing. The five options are: (1) a micro-levy (ie tax tied to submissions of data); (2) subscriptions; (3) cloud-based fees; (4) common licencing and (5) blockchain metadata. Although the proposal offers several options with a range of pros and cons, the options all recognise that the rapidly increasing volume and movement of DSI data are making committing to any one choice more and more difficult.

Meanwhile, the private sector has been producing DSI and creating their own databases. Even as far back as 2002, Syngenta published a new rice genome in the popular academic journal *Science* without making the data available through public open-access databases and instead placing it on their website (see Moore 2002). It is the proliferation of privately owned and managed DSI databases that poses the greatest risk for the Global South over access to and benefit-sharing of genetic resources for food and agriculture. The private sector has little to no interest in the creation of any traceability mechanisms or other tools that could support benefit-sharing and ultimately affect their profits. Current global governance frameworks present no real barriers for the private sector to further produce and access DSI data or to create and own DSI databases. As mentioned above, the Plant Treaty has no legally binding mechanism to enforce payments from private-sector users of the Annex 1 crops, whether they are material resources or DSI. This poses challenges to maintain new or existing DSI in the public domain, independently of whether or not databases are open access.

The inability of the CBD and Plant Treaty to effectively govern DSI leaves open opportunities for the private sector to create and control DSI databases. While individual countries have their own legal challenges in patenting plant genes, the trend of growing private-sector patents continues (Jefferson et al. 2015). The INDSC provides an important starting point for how open-access DSI databases can operate in the best interests of the global public good. Yet, as merely data hosts, the INDSC and its partners do not go far enough in guaranteeing unrestricted free and open access to all. DSI has the potential to serve as a ‘loophole’ that could exacerbate the already growing gulf of funding between public- and private-sector agricultural research. And this divide has important connections with the divide between the Global North and Global South.

The neoliberalisation of nature has a long history of being a largely colonial and postcolonial-driven process by countries currently considered to be in the Global North (Goldman 1998; McCarthy 2004; Castree 2010). Agricultural research has been increasingly undergoing a process of privatisation throughout the twentieth century (Kloppenburg 2004). One of the Contracting Party negotiators I interviewed admitted that the US and Canada are considered by most other Contracting Parties to be heavily influenced by the private sector. The negotiator further mentioned that this influence of the private sector is increasingly favouring the US/Canada position to keep DSI out of the CBD/Plant Treaty frameworks because the current governance arrangement allows companies access to ‘digital genetic material where physical material is no longer needed’ (personal interview, 7 April 2021). In other words, the arrival and rapidly increasing uses of DSI present a new scale of challenges in the global governance of genetic resource exchange (see Laird et al. 2020).

On the one hand, if the CBD and Plant Treaty decide to build a system of access and benefit-sharing for DSI, this could create an additional layer of bureaucracy that would incentivise the private sector to further build their own DSI databases. But, on the other hand, a failure to take multilateral action also risks increased private control over DSI unless guarantees are in place to ensure open access to DSI, from the initial sequencing of a genetic resource (origin) to a range of downstream applications (including patenting). I suggest that an open-source approach offers a pathway for public research to contribute to and draw from the future of DSI for food and agriculture.

## Open-source options for access to and benefit-sharing of digital genetic diversity

Open-source approaches to innovation have origins in the software industry (see Hope 2009). In the case of biotechnology, open-source models were envisioned by scientists and academics as a way to combat what Heller and Eisenberg (1998) called ‘the tragedy of the anticommons’. The ‘anticommons’ concept is based on the proliferation of intellectual property laws in the 1990s which limited innovation due to the privatisation of research and research materials blocking other users from using scarce resources. It is within this context that the Open Source Seed Initiative (OSSI) was established in 2012. The OSSI was developed by a group of academics, plant breeders and farmers who were concerned with large agribusiness companies privatising seeds and other plant genetic material. At the foundation of the OSSI is a pledge that states:

You have the freedom to use these OSSI-Pledged seeds in any way you choose. In return, you pledge not to restrict others’ use of these seeds or their derivatives by patents or other means, and to include this pledge with any transfer of these seeds or their derivatives.

The OSSI pledge operates as a ‘protected commons’ which offers public access to plant, save and improve any cultivar but without limitations on further downstream use. Plant breeders wishing to access or submit seeds are identifiable and providers receive credit for their effort in submitting an improved cultivar. Beyond receiving the credit, Luby and Goldman (2016) explain that ‘OSSI-Pledged seeds can be bred, sold, shared and reproduced as long as any subsequent derivative or reproduction of that seed also carries the same freedoms’ (4). Open-source protection and enforcement can also come in the form of licences, currently being implemented in some European countries under the OSSI framework (see Kotschi and Horneburg 2018). This form of protection is another way to legally protect the open-source commons for all genetic resources and whatever derivatives are generated from them.

OSSI’s ‘protected commons’ approach stands in contrast to other approaches of ‘freely available’ or open-access data. Any attempt to privatise modified varieties accessed from the OSSI is in breach of the open-source guidelines and is then considered ‘copytheft’. This is currently not the case with existing open-access (*libre*) DSI databases. For one, the INSDC serves as a repository of data for researchers willing to submit DSI and allows for open access (*libre*) but the INSDC does not own or control the ownership over the data. There is no way to control what happens with DSI downstream. The Human Genome Project offers another interesting case of the blurred relationship between public and private ownership over sequence data. Celera Genomics, a private company, famously sequenced human DNA in part by relying on data from the Human Genome Project. Celera made their data ‘freely available’ but only under a ‘non-redistribution agreement’ (Nature 2000), which is precisely what the OSSI attempts to prevent: a protected commons that facilitates users to access, improve and redistribute seeds under a pledge or licencing. The OSSI’s reliance on a pledge is more of a strategy to build a movement that embraces and shares open-source approaches due to the limited legal enforcement of open-source licencing worldwide (Kloppenburg 2014).

Another initiative under the ‘protective commons’ approach is the Biological Innovation for Open Society Initiative (BiOS). BiOS was started in 2004 by Cambia, an Australian-based non-profit that has a vision to ‘democratise, decentralise and diversify’ research and research materials (see Berthels 2009). Their approach is to use material transfer agreements (MTAs) as a legal foundation for researchers to share, improve and maintain open access to genetic material. Any researcher who applies BiOS licencing under a MTA agrees to ‘not asset any intellectual property rights to the material and any derivatives and information’ which includes DNA and protein sequences.^5^ Both BiOS and the OSSI present hopeful models to look at productive ways to incorporate DSI under the CBD and Plant Treaty. The former is directed more at researchers whereas the latter aims to limit the privatisation of seeds and to ensure seed saving for farmers. Both are intended to support historically marginalised regions that are facing limited access to genetic resources due to the proliferation of privatisation in the biotechnology sector. However, both BiOS and the OSSI have a relatively limited network. The OSSI was initially established in the US and has only recently gained ground in Europe, due to the limits of national legal frameworks (Kloppenburg 2014). A global approach under the CBD and Plant Treaty would facilitate the scaling up of efforts from the OSSI and BiOS for the benefit of public agricultural research globally.

Adopting a ‘protected commons’ approach for DSI under the CDB and Plant Treaty frameworks could be quite seamless. For example, the Plant Treaty operates a Global Information System (GLIS)^6^ that contains all documentation and information on cultivars available within the MLS (see Ker, Louafi, and Sanou 2013). The GLIS could be expanded to include and trace DSI as it relates to genetic resources for food and agriculture. The Plant Treaty also has the political buy-in of all 164 signing countries. One significant problem with open-access data is that not all countries have the scientific capacity to reap the benefits (Leonelli 2014). But the open-source approach would ensure that all Contracting Parties have equal and unfettered access to DSI and, more importantly, any of the derivatives from it.

## Final considerations

Historical concerns of genetic exploitation by the Global North are also now less relevant than they were during the framing of global governance mechanisms in the twentieth century and up until a few decades ago. Global gene flows have actually reversed. The current concern, therefore, is over the public–private divide that is threatening access to and control over DSI for all. DSI provides new opportunities for the private sector to exploit genetic diversity while simultaneously restricting access and benefit-sharing from the results of research. The public sector, on the other hand, must operate within the existing CBD/Plant Treaty framework to gain access to and share the benefits of genetic resources. This leaves them at a disadvantage. An open-source platform for DSI would benefit all (including the private sector), and at least it would not limit the access to and benefit-sharing of data under the current rules of the CBD and the Plant Treaty.

According to some of the country negotiators I spoke to, the issue of DSI is restructuring the coalitions in the CBD and Plant Treaty. The previous divide in Global North and Global South is getting more complicated as many countries, such as China, Brazil and India, are major users and providers of both physical genetic resources and DSI. Nevertheless, countries in the Global South are largely opposed to the idea of an open-source platform for DSI on a worldwide scale. The reasons negotiators expressed to me were primarily based on the technological capacity – both public and private – to utilise emerging DSI tools. While the current state of private-sector agricultural research as in relates to DSI is beyond the scope of this paper, public-sector research is reliant on the access and benefit-sharing system established by the Nagoya Protocol (CBD) and the Plant Treaty.

There are ongoing concerns over the North–South divide in countries’ technological capacity to exploit new tools such as DSI under an open-source arrangement (Leonelli 2014). But perhaps this divide will only widen should DSI be further privatised, which ostensibly restricts access for the public sector in both the Global North and South. Under the current global governance frameworks DSI is accessed and used as an unprotected commons, meaning anyone can apply for Intellectual Property (IP) and limit downstream innovation. This is one area where further research on the private sector’s role in accessing and benefitting from DSI would shed more light on the public–private divide. Yet for public research, the CBD and Plant Treaty have established parallel systems of restricting access and use to facilitate benefit-sharing. By assuring a ‘protected commons’ of DSI, the CBD and Plant Treaty could help spur innovation in the public sector, improve traceability and globalise existing open-source initiatives by including DSI for food and agriculture. Concerns over the digitisation of biopiracy might better be directed at the current trends in the private sector’s use of DSI. It is for this reason that the public sector and open-source approaches could be brought together to ensure equitable access to the new frontier of digital biodiversity.

## Notes

1 Two thousand of Wickham’s 70,000 seeds were successfully germinated at Kew.

2 Some economically and culturally important crops such as rice, cassava, wheat and maize are included. However, the list is criticised for not including soybeans and for disproportionately including forage crops (see Visser 2013).

3 The MLS intends to do this by creating a unified mechanism for the exchange of plant genetic material under Material Transfer Agreements. However, not all Annex 1 crops operate freely within the MLS via Material Transfer Agreements. Only crops that are “under the management and control of the contracting parties, and are in the public domain” are included within the MLS (Moore and Tymowski 2005, 15). This is why Material Transfer Agreements exist. The agreements subject recipients of germplasm to pay a fee into the Benefit Sharing Fund of the Plant Treaty. A fee is only necessary when the material accessed is used for a result (ie crop variety) that is not then shared back into the MLS, or if the material accessed is used for commercial purposes. The Benefit Sharing Fund is then used to invest in further activities of the Plant Treaty Governing Body as well as national efforts to support public research and farmers from the Global South. From January 2007 until March 2019, more than 4.6 million genetic resource materials have been transferred worldwide through over 68,000 Material Transfer Agreements. And in June 2018 the first user-based payment was received into the Benefit Sharing Fund from a Dutch seed company (FAO 2019).

4 Full factsheet available at: https://www.genome.gov/about-genomics/fact-sheets/Sequencing-Human-Genome-cost.

5 For the full MTA see: https://web.archive.org/web/20090911232408/http://www.bios.net/daisy/bios/3003.html.

6 See: https://ssl.fao.org/glis/.
